# A Toddler's Rehabilitation Following Ilizarov's Tibial Pseudoarthrosis Fixation

**DOI:** 10.7759/cureus.27347

**Published:** 2022-07-27

**Authors:** Tasneem M Lakkadsha, Deepak Jain, Pratik Phansopkar

**Affiliations:** 1 Department of Physiotherapy, Ravi Nair Physiotherapy College, Datta Meghe Institute of Medical Sciences, Wardha, IND

**Keywords:** pediatric rehabilitation, case report, ilizarov’s fixation, physiotherapy, pseudoarthrosis tibia

## Abstract

One of the rare conditions affecting children is pseudoarthrosis of the tibia. The tibia of the affected leg develops a deformity inflicting it to bend backward. We present one such case, who visited the physiotherapy department for post-operative care after receiving Ilizarov's external fixator, which was used to correct this deformity. On presentation, her hip and knee ranges were significantly reduced, her strength in the affected limb had decreased, and her ankle ranges were almost minimal. Her physiotherapy regimen was meticulously planned with consideration for her age and pleasurable activities, allowing us to easily achieve our desired outcome through this play therapy. We noticed significant improvements in her strength and joint ranges after prescribing her routines to follow at home for a month. We thus conclude that physiotherapy is effective in treating this unusual condition.

## Introduction

Congenital pseudoarthrosis of the tibia (CPT) is a condition that can present itself in a variety of ways, ranging from a simple tibial angulation to total non-union with severe bony abnormalities. Type 1 neurofibromatosis and CPT are related, but the causal relation has not been established. Unquestionably significant in the pathophysiology were the fibrous soft tissue and aberrant periosteum seen in pseudoarthrosis, likely as a result of reduced osteogenic potential and altered local vascularization [1]. Its long-term and difficult management poses many challenges. Long-term functionality should continue to be the goal of decision-making. The most notable advancements in care in recent years have come from the use of bone rod constructions, morphogenic proteins, and guided growth in deformity correction. The ability to simultaneously treat lesions of pseudoarthrosis and all of their potential side effects, including leg length discrepancy, refracture, and ankle valgus, is one of the main benefits of an external fixation technique, which was first introduced by Ilizarov in 1971. The benefit of Ilizarov's method is that it can provide a multi-targeted approach; external fixation can be used for distraction, compression, or bone transfer at different levels of the tibia. The complexity and length of the procedure as well as the possibilities for infection are potential drawbacks of the method. Additionally, it has been demonstrated that femoral overgrowth can be brought on by hyperemic distraction osteogenesis [2]. Around 50% of patients who received treatment using Ilizarov's method had positive outcomes [3]. Careful physiotherapy is necessary to prevent joint contractures and subluxations, which in this case occur as a result of muscle irritation brought on by pins or cables impaling them. Stretching and maintaining range of motion is essential for preventing contractures, dislocations, and subluxations, while functional loading and ambulation are required for the ossification of the regenerating bone, clearly stating how crucial physical therapy is to the success of Ilizarov's procedure [4].

## Case presentation

Patient information

**Figure 1:**
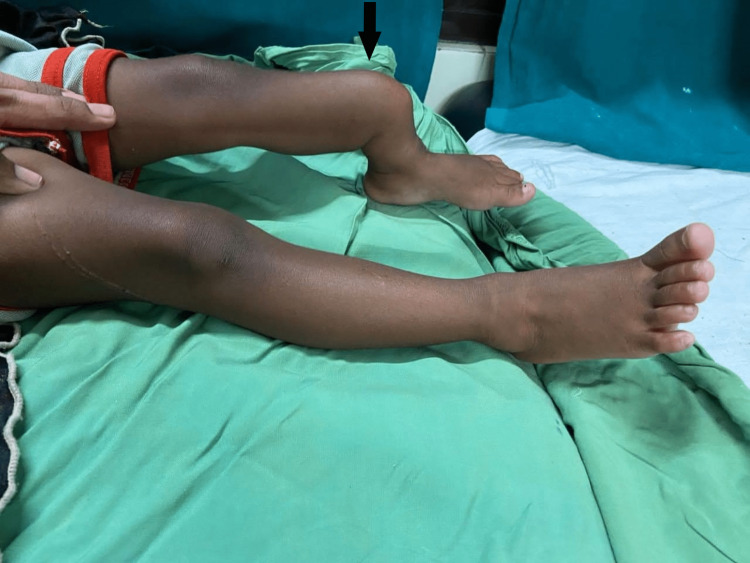
The day patient presented to the orthopedic department The black arrow shows the site of the lesion over the left tibia

**Figure 2:**
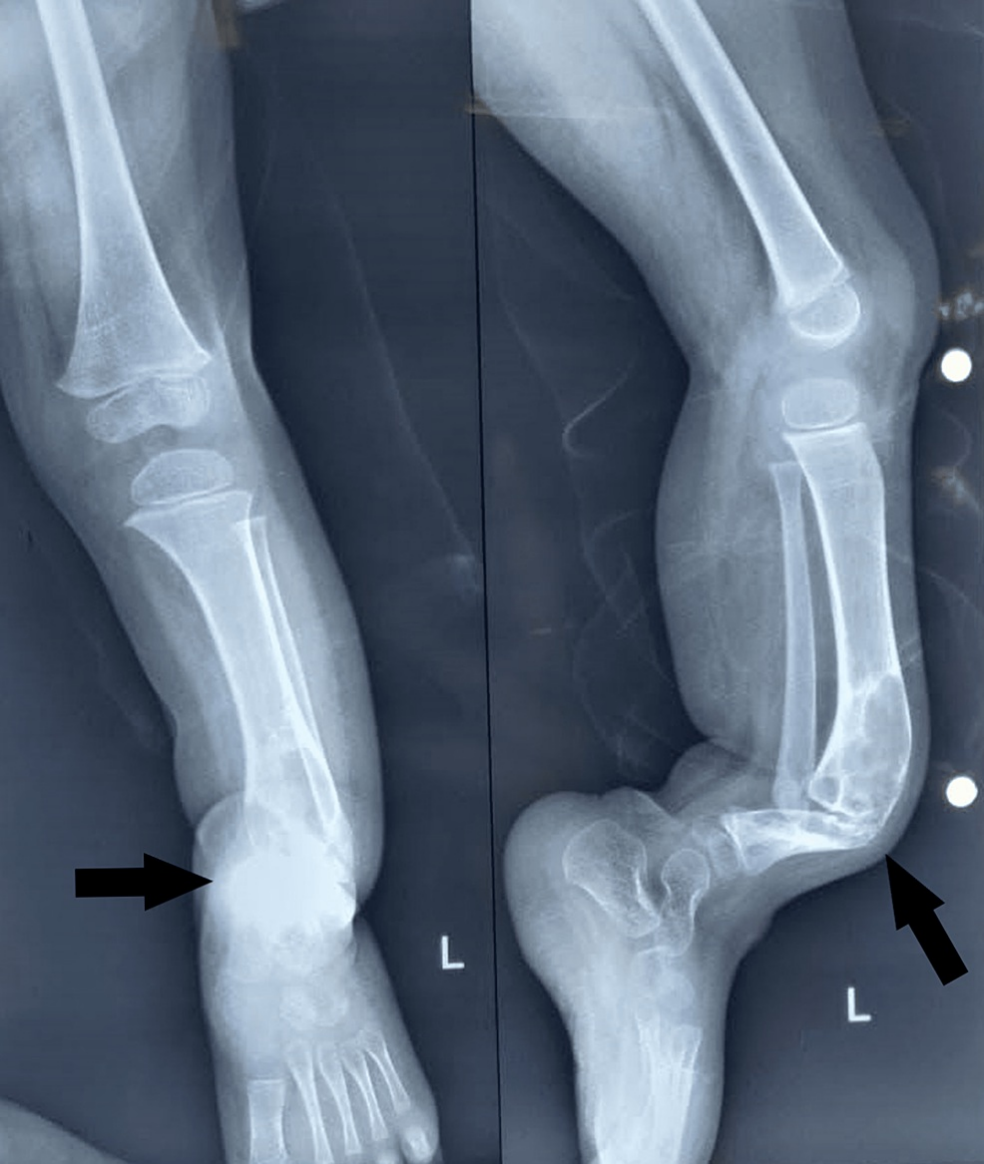
X-ray of the left tibia anteriorly (L) and laterally (R) showing pseudoarthrosis with signs of sclerosis The black arrows show the site of lesion on the tibia over the X-rays

A two-year-old girl with a deformed leg and obvious gait abnormality came in with her parents to the orthopedic department (Figure 1). It had disturbed her daily chores and activities. Her X-rays (Figure 2) and other investigations indicated that she had a congenital pseudoarthrosis of the left tibia. She was her parents’ first by order and had no family history of any relevant conditions. For the same reason, she underwent surgery shortly afterward; a wedge osteotomy and an external Ilizarov's fixation were performed. She was transferred to the physiotherapy department for additional management after a week.

**Figure 3:**
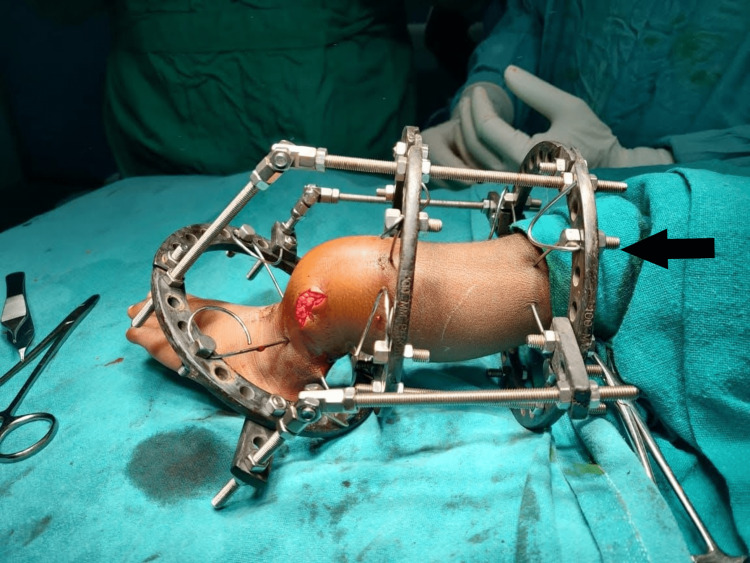
Left lower limb is seen with external fixation

On presentation, she was examined thoroughly in supine lying. Except for the inability to ambulate or perform tasks that required weight-bearing on both limbs, her developmental milestones were achieved on time, and she was conscious, alert, cooperative, thin-built, and vitally stable. There was a localized deformity, i.e., her left tibia was bent backward in the distal part and an Ilizarov’s fixation was seen over it. There was only an open wound present in that region, with no discernible muscle wasting, discoloration, edema, swelling, or temperature increase (Figure 3).

A distinct spasm around the tibia in the left leg muscles was palpable during the movement examination. The range and end feel of the joints in the right lower limb were within normal limits. The range of motion in the left knee was reduced considerably and nearly lost at the ankle. The approximate ranges of the left lower limb are depicted in Table 1. On the left side, the knee and hip felt firm, but the ankle joint felt hard and bony. Manual muscle testing scores on the right side were slightly lower, while those on the left were significantly lower (Table 2). There was a true shortening of the left lower limb with an 8-cm discrepancy from the corresponding other. The girth of the thigh was equal on both sides in the left lower leg, but the girth of the leg was different due to the sheer bulking of the muscle at a location because of the deformity and potential muscle wasting.

**Table 1:** Range of motion of left lower limb joints ROM, range of motion

Joint	Movement	ROM of the left lower limb	
		Active	Passive
Ankle	Plantarflexion	0°	5°
	Dorsiflexion	0°	0°
	Inversion	0°	0°
	Eversion	0°	0°
Knee	Flexion	90°	90°
	Extension	0°	0°
Hip	Flexion	90°	90°
	Extension	10°	15°
	Internal rotation	10°	15°
	External rotation	15°	20°
	Abduction	30°	40°
	Adduction	10°	15°

**Table 2:** Manual muscle test grading

Joint	Muscles	Grading	
		Left	Right
Ankle	Plantar flexors	2	3
	Dorsiflexors	1	3
	Invertors	1	3
	Evertors	1	3
Knee	Flexors	3	3
	Extensors	3	3
Hip	Flexors	3	3
	Extensors	3	3
	Internal rotators	3	3
	External rotators	3	3
	Abductors	3	3
	Adductors	3	3

Therapeutic interventions

Her regimen was created with consideration for her age. After a thorough discussion with her parents, the treatment's objectives were formulated. They received counselling on how to implement the home exercise program, as well as on the methods, length, and expectations for the physiotherapy treatment. Table 3 lists her objectives and the required actions to achieve each one. There was an unpredictability of the recovery time needed to restore the limb to normal function; hence, the intervention was continued indefinitely. As a result, it was anticipated that the regimen was to be followed until the end of the orthopedic treatment. Each intervention was performed five times per day, progressing as the physiotherapist sees fit.

**Table 3:** Physiotherapy intervention

Sr. no.	Goals	Physiotherapy intervention	Rationale
1.	Pain reduction	Ice massage	Ice reduces the pain by numbing the area, i.e., action through the pain gait mechanism.
2.	To reduce or prevent limb edema	Elevation and toe movements	Gravity assists with edema drainage in elevation and toe movement facilitates the same.
3.	To prevent or relieve muscles and fascia tightness	Myofascial release of the plantar flexors	This loosens up the muscles and fascia and prevents them from going into tightness.
		Positioning	Positioning of the knees during sleep using pillows to overcome the fixator’s height and prevent strain over the structures of the knee and hip.
4.	Providing patient proprioceptive sensations	Pressure over the sole of the foot to compress the joints of that limb	This was used to help her to gain a sense of proprioception in the initial stages when she could not stand on the frame.
		Making the patient stand on a walking frame	While doing this activity, she gained proprioception through the non-affected extremity and through the affected extremity when it touched the ground.
		Proprioceptive neuromuscular facilitation	All the diagonal patterns were practiced upon her initially and later by herself to gain the customary memory for the muscles to perform the daily living activities.
5.	Gaining or maintaining normal range of motion of the adjacent joints, i.e., hip, knee, and possibly ankle	Kicking ball while standing on the walking frame in every direction	Since the patient was a child, she could not understand the commands of the therapist; hence, the hip and knee ranges were actively maintained as well as improved with play therapy.
		Hydrotherapy	When the child was immersed in the pool, she moved her limbs by herself to enjoy the water and thus we gained the range of every joint effortlessly.
6.	Maintaining the strength of the muscles of adjacent joints	Make the child squeeze a sponge ball below the buttocks, thighs, and Ilizarov’s fixation	To further increase the strength of the limb, isometrics were added this way.

Follow-up and outcome of interventions

Every month, the patient was called for a follow-up appointment to assess how her regimen was progressing. The range of motion in the knee, ankle, and foot, as well as manual muscle testing of the main muscles surrounding the joint, were used as outcome measures for evaluation at one month. From the first day of evaluation, both outcome measures significantly improved (Tables 1, 2). The manual muscle testing grade for the muscles that were graded 3 on day 1 was grade 3+ (Table 2), but the muscles with grades 1 and 2 remained unchanged. Overall, there was a gross 5° improvement in the ranges.

## Discussion

For the duration of the frame's use, an Ilizarov's external fixator-stabilized limb must be used physiologically. Weight-bearing for the lower extremity and functional use of the upper limbs are necessary for proper growth and ossification. Myofascial tissues resist elongation during any Ilizarov's surgery involving bone segment movement, whether it is lengthening, deformity repair, or bone transport, which can result in joint contractures that can lead to subluxations and dislocations or progressive deformity at the site of a corticotomy. As a result, intensive physical therapy, dynamic and static splinting, and ideal sleeping positions must be used throughout the patient's fixation [4]. According to a study, early weight bearing and range of motion exercises should be prioritized because it does not seem beneficial to remain immobile [5]. We trained this child in the water for both strength and range through a 1999 study that examined the use of hydrotherapy in Ilizarov's fixator as beneficial [6]. Overall, the majority of physical therapy techniques are fundamental, such as icing, hydrotherapy, and myofascial release, but some have been tailored to her age, such as kicking a ball through the injured limb while supporting weight on the unaffected limb on a standing frame, or squeezing a sponge ball under different parts of the lower limb, such as below the thigh or leg for isometric strengthening.

## Conclusions

For a child with pseudoarthrosis of the tibia recovering from and using an Ilizarov's fixator, physical therapy is crucial. It is clear from the improvement in the outcome measures that the patient's joint ranges and strength have improved since the evaluation day. These help her reach the developmental milestone of being able to stand, walk, and run on her own.

## References

[1] Pannier S (2011). Congenital pseudarthrosis of the tibia. Orthop Traumatol Surg Res.

[2] Eisenberg KA, Vuillermin CB (2019). Management of congenital pseudoarthrosis of the tibia and fibula. Curr Rev Musculoskelet Med.

[3] Javaid MZ, Khan J, Javaid MM, Zafar Z, Shakir IA, Bashir S (2020). Outcome of congenital pseudoarthrosis tibia treated with Illizarov fixator in children. Int. J. of Orth.

[4] Green Green, Stuart A (1990). Physiotherapy during Ilizarov fixation. Tech Orthop.

[5] Kabst C, Tian X, Kleber C, Amlang M, Findeisen L, Lee G, Zwingenberger S (2022). Prolonged application of continuous passive movement improves the postoperative recovery of tibial head fractures: a prospective randomized controlled study. Biomed Res Int.

[6] Barker K, Burns M, Littler S (1999). Physiotherapy for patients with an Ilizarov external fixator: a survey of current practice. Physiotherapy.

